# Timing of symptomatic intracranial hemorrhage after endovascular stroke treatment

**DOI:** 10.1177/23969873221112279

**Published:** 2022-08-03

**Authors:** Wouter van der Steen, Nadinda AM van der Ende, Katinka R van Kranendonk, Vicky Chalos, Josje Brouwer, Robert J van Oostenbrugge, Wim H van Zwam, Pieter J van Doormaal, Adriaan CGM van Es, Charles BLM Majoie, Aad van der Lugt, Diederik WJ Dippel, Bob Roozenbeek

**Affiliations:** 1 Department of Neurology, Erasmus MC University Medical Center, Rotterdam, The Netherlands; 2 Department of Radiology and Nuclear Medicine, Erasmus MC University Medical Center, Rotterdam, The Netherlands; 3 Department of Radiology and Nuclear Medicine, Amsterdam University Medical Centers, Location AMC, Amsterdam, The Netherlands; 4 Department of Public Health, Erasmus MC University Medical Center, Rotterdam, The Netherlands; 5 Department of Neurology, Amsterdam University Medical Centers, Location AMC, Amsterdam, The Netherlands; 6 Department of Neurology, Maastricht University Medical Center, Cardiovascular Research Institute Maastricht (CARIM), Maastricht, The Netherlands; 7 Department of Radiology and Nuclear Medicine, Maastricht University Medical Center, Cardiovascular Research Institute Maastricht (CARIM), Maastricht, The Netherlands; 8 Department of Radiology, Leiden University Medical Center, Leiden, The Netherlands

**Keywords:** Ischemic stroke, endovascular therapy, symptomatic intracranial hemorrhage, timing, surveillance time points

## Abstract

**Introduction:**

Little is known about the timing of occurrence of symptomatic intracranial hemorrhage (sICH) after endovascular therapy (EVT) for acute ischemic stroke. A better understanding could optimize in-hospital surveillance time points and duration. The aim of this study was to delineate the probability of sICH over time and to identify factors associated with its timing.

**Patients and methods:**

We retrospectively analyzed data from the Dutch MR CLEAN trial and MR CLEAN Registry. We included adult patients who underwent EVT for an anterior circulation large vessel occlusion within 6.5 h of stroke onset. In patients with sICH (defined as ICH causing an increase of ⩾4 points on the National Institutes of Health Stroke Scale [NIHSS]), univariable and multivariable linear regression analysis was used to identify factors associated with the timing of sICH. This was defined as the time between end of EVT and the time of first CT-scan on which ICH was seen as a proxy.

**Results:**

SICH occurred in 205 (6%) of 3391 included patients. Median time from end of EVT procedure to sICH detection on NCCT was 9.0 [IQR 2.9–22.5] hours, with a rapidly decreasing incidence after 24 h. None of the analyzed factors, including baseline NIHSS, intravenous alteplase treatment, and poor reperfusion at the end of the procedure were associated with the timing of sICH.

**Conclusion:**

SICHs primarily occur in the first hours after EVT, and less frequently beyond 24 h. Guidelines that recommend to perform frequent neurological assessments for at least 24 h after intravenous alteplase treatment can be applied to ischemic stroke patients treated with EVT.

## Introduction

The guidelines of the American Heart Association and American Stroke Association recommend to “perform neurological assessments every 15 min during and after intravenous alteplase infusion for 2 h, then every 30 min for 6 h, then hourly until 24 h after intravenous alteplase treatment.”^1^ The main reason for these regular neurological assessments is early recognition of a possible symptomatic intracranial hemorrhage (sICH) occurring during and after intravenous alteplase infusion.^2^ As there are no specific recommendations for ischemic stroke patients treated with endovascular therapy (EVT), these recommendations are often extrapolated to this patient population. However, the timing patterns of sICH occurrence after EVT may differ from those after intravenous alteplase treatment.

The 24-h window is primarily based on results of the pivotal NINDS trial, in which 95% of sICHs occurred within 24 h and 50% occurred during the first 8 h.^3^ However, this study was performed in the pre-EVT era, and only 22 sICHs occurred in 623 included patients (20 in 311 patients treated with intravenous alteplase, and 2 in 312 patients treated with placebo). A good understanding of the timing patterns of sICH after EVT with or without prior intravenous alteplase treatment is missing. Such understanding could optimize surveillance time points and duration of in-hospital surveillance in patients treated with EVT. In addition, an understanding of patient characteristics associated with the timing of sICH after EVT could guide the selection of patients in need of stricter or longer surveillance and repeated brain imaging.

The aim of this study was to delineate the probability of sICH occurrence over time in patients treated with EVT after an anterior large vessel occlusive stroke and to identify factors associated with the timing of sICH.

## Patients and methods

### Study design and patients

We retrospectively analyzed data from the Multicenter Randomized Clinical Trial of Endovascular Treatment for Acute Ischemic Stroke in the Netherlands (MR CLEAN trial) and the MR CLEAN Registry. The MR CLEAN trial was a phase III multicenter clinical trial with randomized treatment group assignment, open label treatment, and blinded outcome evaluation. EVT plus usual care (intervention group) was compared with usual care alone (control group). Patients were enrolled between December 2010 and March 2014. The MR CLEAN Registry was a national, prospective, open, multicenter, observational monitoring study for stroke intervention centers that perform EVT in the Netherlands. It includes patients with ischemic stroke who underwent EVT since the completion of the MR CLEAN trial. We used verified data at the time of analysis, which includes data of all patients registered between March 2014 and November 2017. Details on both the MR CLEAN trial and the MR CLEAN Registry were published previously.^4–6^

For the current analysis, we included adult patients who underwent EVT (defined as entry into the angiography suite and undergoing arterial puncture), were treated in a center that participated in the MR CLEAN trial, had a proximal intracranial large vessel occlusion in the anterior circulation (internal carotid artery (ICA), internal carotid artery terminus (ICA-T), middle (M1/M2/M3) cerebral artery, or anterior (A1/A2) cerebral artery), and had an onset to groin puncture time of <6.5 h. Patients with a large vessel occlusion in the posterior circulation were excluded, because they have different etiology, pathology, and ICH risks than anterior circulation strokes.^7,8^

### Outcomes

In both the MR CLEAN trial and the MR CLEAN Registry, sICH was defined as neurological deterioration (an increase of four or more points on National Institutes of Health Stroke Scale (NIHSS)) and ICH detected on follow-up imaging (NCCT or MRI) within 3 months after EVT being judged to be the cause of the clinical deterioration. ICH could include hemorrhagic infarctions, parenchymal hematomas, and hemorrhages outside infarcted brain tissue (i.e. subarachnoid hemorrhage, intraventricular hemorrhage, subdural hemorrhage, or remote parenchymal hemorrhage). Follow-up imaging was assessed by an imaging core laboratory and discharge letters were assessed for neurological deterioration by the research coordinators. A serious adverse event committee assessed the combined information to determine whether or not sICH occurred. If neurological deterioration occurred but follow-up imaging could not be obtained by the research group, the serious adverse event committee assessed information from the discharge letter including course of the admission and reports of local imaging assessments to determine whether or not sICH occurred. The time of neurological deterioration (i.e. sICH occurrence) was not documented. However, in the Netherlands it is standard protocol to perform a CT-scan immediately after neurological deterioration occurs. Therefore, the timing of sICH was defined as the time between end of endovascular procedure and the examination time of the first CT scan on which ICH was seen as a proxy.

### Statistical analysis

We presented the baseline clinical, radiological and treatment-related characteristics of our study population stratified by the timing of sICH (categorized at 0–24 h/⩾24 h/missing and no sICH occurrence). We used median and interquartile range (IQR) for continuous variables and frequencies and percentages for categorical variables. Additionally, we presented the cumulative probability plot with censoring for deceased patients and patients lost to follow-up, and a barplot of the timing and frequency of sICH occurrence during the first 5 days after EVT. We presented both plots with imputed data of missing values for time to sICH.

In the subset of patients with sICH occurrence, we used multivariable linear regression analysis to identify characteristics that are associated with the timing of sICH. As the data of timing of sICH was skewed to the right we used a logarithmic scale for the analysis. To avoid strong influence of outliers, we truncated all timings of sICHs above the 95th percentile with the value of the 95th percentile. We selected clinical, radiological or treatment-related factors based on literature (i.e. risk factors sICH) and expert opinion. To ensure that the linear regression analyses had sufficient statistical power, we restricted the number of evaluated independent variables to one per every 10 subjects with sICH. Evaluated characteristics included age; sex; history of stroke; history of atrial fibrillation; history of other vascular disease (i.e. hypertension, hypercholesterolemia, diabetes mellitus, myocardial infarction, or peripheral arterial disease); prior use of antiplatelets; prior use of anticoagulants (i.e. direct oral anticoagulant, coumarin or heparin); NIHSS at baseline; systolic blood pressure at baseline; glucose level; platelet count; International Normalized Ratio (INR); Alberta Stroke Program Early CT Score (ASPECTS) at baseline; poor collateral score (<50%); treatment with intravenous alteplase; performed procedure (thrombectomy vs catheterization or digital subtraction angiography [DSA] only); poor reperfusion measured with the post-EVT modified treatment in cerebral ischemia (mTICI ⩽ 2A) score; and onset to reperfusion time. Results were presented as beta-coefficients (β) with 95% confidence intervals.

All statistical analyses were performed with R version 4.0.5 (www.cran.r-project.org), with the packages: *Hmisc, rms, tableone, dplyr*. For regression analyses, we replaced missing values for independent variables with multiple imputation (*n* = 5 imputation sets) using the aregImpute function. In addition, we performed a sensitivity analysis including imputation of missing values for time to sICH. Residual plots and QQ plots were used to visually check homoscedasticity and normality assumptions. The data of the MR CLEAN trial have been made publicly available at the Virtual International Stroke Trials Archive and can be accessed at http://www.virtualtrialsarchives.org/vista/. Individual patient data of the MR CLEAN Registry cannot be made available under Dutch law, as we did not obtain patient approval for sharing individual patient data, even in coded form. However, all syntax files and output of statistical analyses will be made available upon reasonable request.

## Results

### Patients

**Figure 1.**
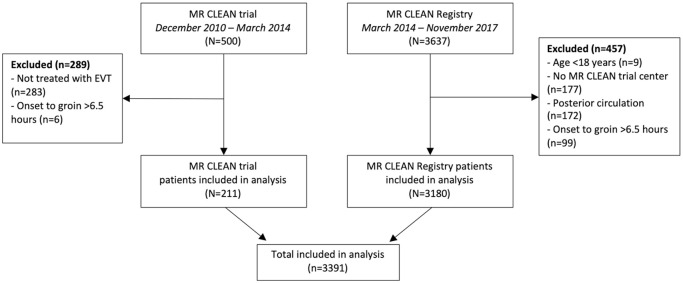
Flowchart of included patients. EVT: endovascular treatment.

Overall, 500 patients were included in the MR CLEAN trial between December 2010 and March 2014, and 3637 patients were registered in the MR CLEAN Registry until November 2017. For this analysis, we excluded 289 patients of the MR CLEAN trial who did not undergo EVT (*n* = 283) or had an onset to groin puncture time of >6.5 h (*n* = 6) (Figure 1). We excluded 457 patients of the MR CLEAN Registry who were aged under 18 years (*n* = 9), had no treatment in a MR CLEAN trial center (*n* = 177), had an intracranial occlusion of the posterior circulation (*n* = 172), or had an onset to groin puncture time exceeding 6.5 h (*n* = 99). In total, 3391 patients remained for the analysis.

### Patient characteristics

Median age was 72 [IQR 61–80] years, 1778 patients (52%) were men, and the median baseline NIHSS was 16 [IQR 11–20] (Table 1). Most included patients had an M1 occlusion (58%), followed by an ICA or ICA-T occlusion (26%) and an M2 occlusion (15%). Median ASPECTS was 9 [IQR 7–10], and 2611 patients (77%) received intravenous alteplase prior to EVT. In total, 2259/61,038 (3.7%) data points of the evaluated patient characteristics were missing.

**Table 1.** Patient characteristics stratified by sICH occurrence, and by timing of sICH after endovascular stroke treatment.

	Timing of sICH <24 h (*n* = 137)	Timing of sICH ⩾24 h (*n* = 41)	Timing of sICH unknown (*n* = 27)	No sICH (*n* = 3186)	Missing
Clinical characteristics					
Age in years; median (IQR]	73 [64–80]	71 [62–83]	73 [66–85]	72 [61–80]	0
Men, n (%)	62 (45)	21 (51)	12 (44)	1683 (53)	0
Pre-stroke mRS score, n (%)					72
0	82 (61)	27 (66)	16 (62)	2155 (69)	
1	25 (19)	5 (12)	1 (3.9)	397 (13)	
2	10 (7.5)	4 (10)	2 (7.7)	225 (7.2)	
>2	17 (13)	5 (12)	7 (27)	341 (11)	
Medical history					
Ischemic stroke, n (%)	24 (18)	3 (7.7)	5 (19)	527 (17)	27
Atrial fibrillation, n (%)	23 (17)	12 (31)	11 (41)	770 (24)	42
Vascular disease, n (%)	102 (77)	27 (68)	20 (74)	2039 (65)	68
Prior antithrombotic drug use					
Antiplatelet, n (%)	65 (49)	16 (39)	7 (27)	953 (30)	41
Direct oral anticoagulant, n (%)	1 (0.8)	0 (0.0)	0 (0.0)	103 (3.5)	249
Coumarine, n (%)	10 (7.4)	7 (17)	7 (26)	401 (13)	24
Heparin, n (%)	4 (3.0)	2 (4.9)	1 (3.9)	98 (3.1)	43
Current smoking, n (%)	27 (26)	8 (28)	5 (24)	696 (28)	740
NIHSS score at baseline; median [IQR]	17 [13–21]	18 [14–20]	16 [14–19]	16 [11–20]	52
SBP at baseline, mmHg; median [IQR]	160 [140–174]	154 [140–168]	155 [147–170]	148 [130–165]	88
Baseline blood levels					
INR; median [IQR]	1.0 [1.0–1.1]	1.0 [1.0–1.1]	1 [1.0–1.2]	1.0 [1.0–1.1]	26
Trombocyte count (10^9^/L); median [IQR]	245 [196–297]	247 [201–281]	229 [198–291]	233 [192–287]	446
Glucose level (mmol/L); median [IQR]	7.7 [6.5–9.4]	7.8 [6.4–10]	7.0 [6.0–8.9]	6.7 [5.9–8.0]	373
Radiological characteristics					
Level of occlusion on CTA, n (%)					126
ICA or ICA-T	40 (30)	16 (39)	9 (35)	785 (26)	
M1	68 (52)	20 (49)	13 (50)	1803 (59)	
M2	23 (17)	4 (10)	4 (15)	446 (15)	
Other (M3/anterior/none)	1 (0.8)	1 (2.4)	0 (0.0)	33 (1.0)	
ASPECTS on NCCT; median [IQR]	9 [7–10]	9 [8–10]	8 [7–9]	9 [7–10]	106
Poor collateral score <50%, n (%)	70 (56)	19 (48)	14 (56)	1226 (41)	204
Treatment-related characteristics					
Intravenous alteplase treatment, n (%)	110 (80)	34 (83)	16 (59)	2451 (77)	11
Performed endovascular procedure, n (%)					220
Catheterization only (no access)	7 (5.6)	1 (2.6)	1 (4.2)	178 (6.0)	
DSA only (spontaneous reperfusion)	7 (5.6)	4 (11)	0 (0.0)	273 (9.2)	
Endovascular treatment	112 (89)	33 (87)	23 (96)	2532 (85)	
Post-EVT mTICI score, n (%)					102
0	29 (21)	7 (18)	6 (22)	505 (16)	
1	5 (3.7)	1 (2.6)	6 (22)	84 (2.7)	
2A	31 (23)	10 (26)	7 (26)	578 (19)	
2B	36 (27)	13 (34)	1 (3.7)	977 (32)	
3	34 (25)	7 (18)	7 (26)	945 (31)	
Time from onset to reperfusion in minutes; median [IQR]	268 [225–328]	279 [225–348]	281 [232–338]	254 [200–317]	219

Continuous variables are presented as median and interquartile range (IQR) or mean and standard deviation (SD). Categorical variables are presented as frequencies (n) and percentages (%). sICH: symptomatic Intracranial Hemorrhage; mRS: modified Rankin Scale; NIHSS: National Institutes of Health Stroke Scale; SBP: systolic blood pressure; INR: International Normalized Ratio; CTA: CT angiography; ICA(-T): internal carotid artery (terminus); M(segment): middle cerebral artery; ASPECTS: Alberta Stroke Program Early CT score; NCCT: Non-Contrast CT; DSA: Digital Subtraction Angiography; EVT: Endovascular Treatment; mTICI: modified Thrombolysis in Cerebral Infarction.

### Outcomes

**Figure 2.**
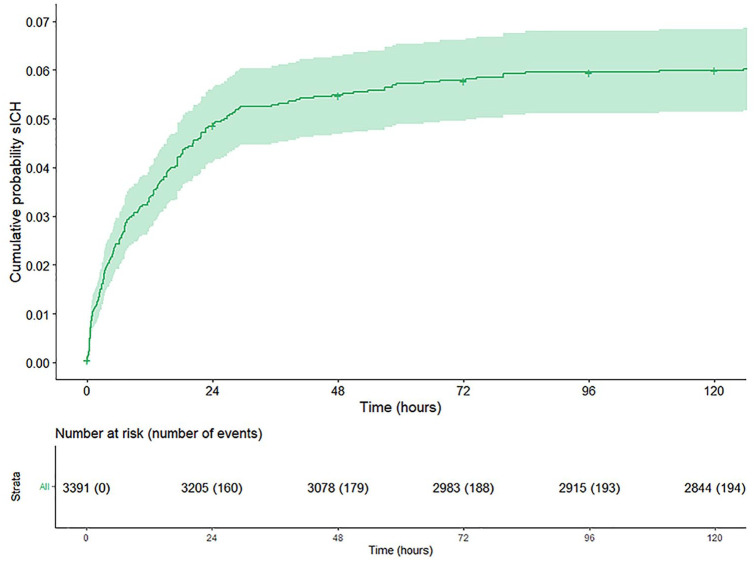
Cumulative probability plot of symptomatic intracranial hemorrhage (sICH) in the first 5 days after endovascular stroke treatment with censoring of deceased patients and patients lost to follow-up. Missing values for time to sICH (27/205) were imputed.

**Figure 3.**
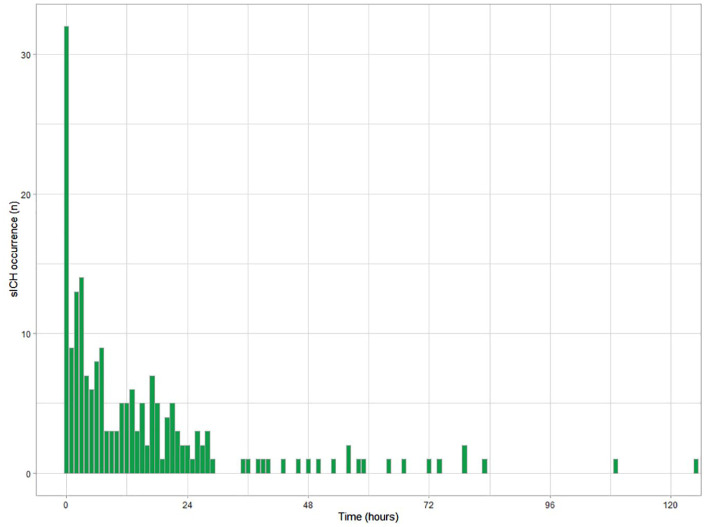
Barplot of the frequency of symptomatic intracranial hemorrhage (sICH) occurrence per hour in the first 5 days after endovascular stroke treatment. Missing values for time to sICH (27/205) were imputed.

SICH occurred in 205/3391 (6%) patients. Median time from end of endovascular procedure to sICH detection on NCCT was 9.0 [IQR 2.9–22.5] hours, with the 95th percentile at 129.5 h. In 98/205 (48%) patients sICH occurred within 12 h, 39/205 (19%) patients between 12 and 24 h, in 12/205 (6%) patients between 24 and 36 h, and in 29/205 (14%) patients between 36 h and 3 months. In 5/205 patients, ICH was also seen on DSA during the EVT. In 27/205 (13%) patients, data on timing of sICH occurrence could not be retrieved as either the time of end of endovascular procedure or the examination time of first CT scan on which sICH was found was missing. After multiple imputation of missing data and with censoring of deceased patients and patients lost to follow-up, the frequency of sICH decreased from 160/3391 (4.7%) in the first 24 h to 19/3205 (0.6%) between 24 and 48 h, to lower frequencies thereafter (Figures 2 and 3). Hemorrhage types found in patients with sICH are given in the supplements (Supplemental Table 1).

**Figure 4.**
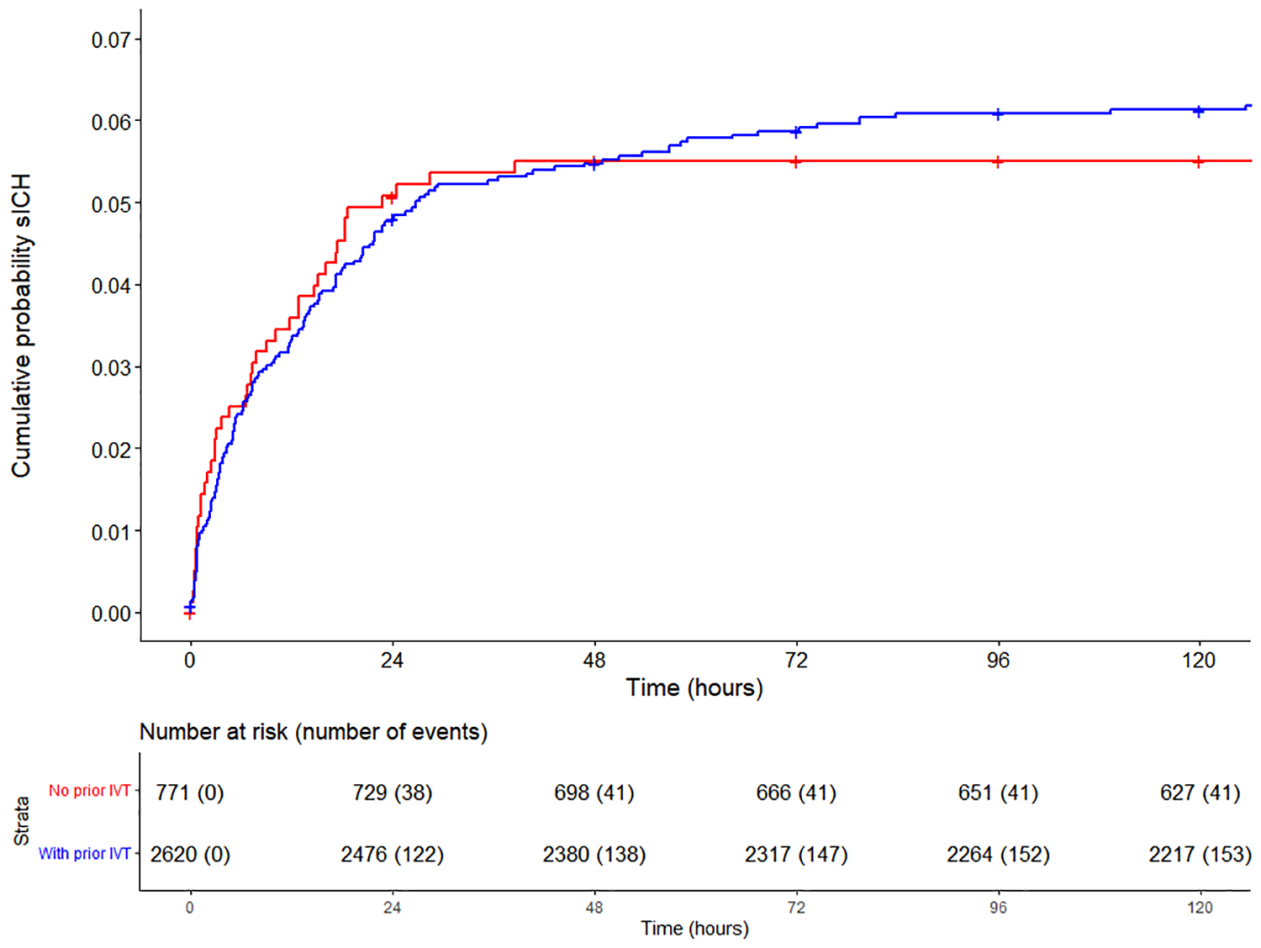
Cumulative probability plot of symptomatic intracranial hemorrhage (sICH) in the first 5 days after endovascular stroke treatment with censoring of deceased patients and patients lost to follow-up. Lines are stratified for no prior treatment with intravenous thrombolysis (IVT; red line) and with prior treatment with IVT (blue line). Missing values for time to sICH (27/205) were imputed.

In patients without prior treatment with intravenous alteplase, sICH occurred in 45/769 (6%) of patients, and median time to sICH detection was 7.5 [IQR 2.2–18.1] hours. This did not differ from patients treated with intravenous alteplase before EVT, in whom sICH occurred in 160/2611 (6%) patients, and median time to sICH detection on NCCT was 9.8 [IQR 3.1–22.9] hours (Figure 4).

### Determinants of timing of sICH occurrence

In both univariable and multivariable regression analysis none of the evaluated characteristics, including age, baseline NIHSS, baseline glucose level, intravenous alteplase treatment, and poor reperfusion at the end of the procedure were associated with the timing of sICH occurrence (Table 2). Sensitivity analyses, including imputation of missing values for time to sICH, showed comparable results (Supplemental Table 2).

**Table 2.** Univariable and multivariable regression analysis of determinants of timing of sICH after endovascular stroke treatment.

Variables	β (95% CI)	Adjusted β (95% CI)
Age (per 10 years)	1.03 (0.80–1.32)	0.88 (0.65–1.20)
Male sex	1.12 (0.61–2.05)	0.85 (0.44–1.65)
Medical history of stroke	0.65 (0.28–1.51)	0.52 (0.19–1.41)
Medical history of atrial fibrillation	1.76 (0.83–3.74)	1.45 (0.55–3.83)
Medical history of vascular disease	1.16 (0.57–2.37)	0.98 (0.44–2.20)
Prior use of antiplatelets	0.97 (0.53–1.78)	1.33 (0.64–2.78)
Prior use of anticoagulants	2.03 (0.80–5.17)	1.88 (0.55–6.47)
Baseline NIHSS (per point increase)	1.03 (0.98–1.09)	1.05 (0.99–1.11)
Baseline systolic blood pressure (per 10 mmHg)	1.02 (0.92–1.15)	1.05 (0.92–1.19)
Baseline glucose level (per mmol/L)	1.00 (0.96–1.04)	0.97 (0.92–1.01)
Baseline platelet count (per 10 ×10^9^/L)	1.00 (0.96–1.03)	1.00 (0.96–1.04)
Baseline INR (per point increase)	2.20 (0.86–5.59)	2.54 (0.73–8.92)
Baseline ASPECTS on NCCT (per point increase)	0.92 (0.79–1.08)	0.87 (0.73–1.04)
Poor collateral score (<50%)	0.89 (0.49–1.65)	0.82 (0.42–1.60)
Intravenous alteplase treatment	0.99 (0.46–2.13)	1.68 (0.66–4.26)
Performed procedure (EVT vs DSA or catheterization only)	0.64 (0.25–1.64)	0.52 (0.19–1.41)
Poor post-EVT mTICI score (<2B)	0.68 (0.37–1.25)	0.59 (0.31–1.11)
Onset to reperfusion time (per hour)	1.00 (1.00–1.01)	1.00 (1.00–1.01)

Univariable and multivariable regression coefficients are presented as beta (β) coefficients with 95% confidence intervals (CI). sICH: symptomatic intracranial hemorrhage; NIHSS: National Institutes of Health Stroke Scale; INR: International Normalized Ratio; ASPECTS: Alberta Stroke Program Early CT score; NCCT: Non-Contrast CT; EVT: Endovascular Therapy; DSA: Digital Subtraction Angiography; mTICI: modified Thrombolysis in Cerebral Infarction.

## Discussion

In this large retrospective study of two combined databases, we found that the frequency of sICH occurrence was highest during the first hours after EVT, after which the frequency rapidly decreased over time. We did not find any characteristics associated with the timing of sICH.

The timing patterns of sICH after EVT found in this study are for a large part in line with timing patterns of sICH found in the NINDS trial, and also with that of a more recent prospective study on the timing of sICH after intravenous alteplase.^3,9^ However, we did find a slightly higher risk of sICH after more than 24 h. This could very well be attributed to the different definitions (neurological deterioration ⩾4 points on the NIHSS vs any neurological deterioration) and time windows (3 months vs 36 h) used for sICH. In line with other observational studies, we found that the majority of sICHs occurred within 12 h after stroke treatment.^2,9,10^ Therefore, more frequent neurological assessments are warranted during these hours. Altogether, it seems justified to extrapolate current protocols on the frequency of neurological assessments after intravenous alteplase treatment to patients treated with EVT. Of note, we only evaluated the timing of sICH, whereas other causes of early neurological deterioration (e.g. reocclusion, infarct extension, cerebral edema, seizures) also warrant frequent neurological assessments.^11^ Other studies should investigate the timing patterns of these complications.

To further improve protocols, selecting patients for which frequent neurological assessments after more than 12 h are not of additional value could reduce workload, length of stay, and hospital costs.^10,11^ In addition, it could be helpful to select patients which warrant stricter and longer neurological assessments. Therefore, we evaluated a potential association with various characteristics and the timing of sICH occurrence. However, we did not find any associations. This could be due to the restricted number of potential characteristics we could evaluate with sufficient statistical power.^12^ Other potential characteristics should be evaluated in different cohorts. These should include established risk factors for sICH occurrence after EVT.^13^ In addition, evaluating post-procedural factors (e.g. systolic blood pressure in the first hours after EVT and initiation of antithrombotic agents) could be more informative, especially because these are parameters we could modify in order to decrease the incidence of sICH.^14^ However, it should be noted that it may be hard to draw definitive conclusions from the analyses of post-procedural factors due to the possibility of reversed causality.

In line with results of randomized controlled trials evaluating the efficacy and safety of bridging IVT prior to EVT, we did not find a difference in the occurrence of sICH in patients with or without prior treatment with intravenous alteplase.^15–18^ Moreover, we found no difference in the timing of sICH between the two groups, and in multivariable regression analysis prior treatment with intravenous alteplase was not associated with the timing of sICH. Alteplase has a short plasma half-life of approximately 4 min, suggesting that its main influence on the risk of ICH would be in the first hours after infusion.^19^ However, the fibrinolytic activity can reduce fibrinogen levels for more than 24 h after completion of the alteplase infusion and the half-life of the fibrin-alteplase complex is not well known.^20^ Prolonged abnormal fibrinogen levels have been associated with a higher risk of ICH.^21^

The results of this study may also be helpful in the design for studies evaluating the optimal timing of initiating or re-initiating antithrombotic agents. The risk of antithrombotic therapy within 24 h after stroke treatment is still uncertain.^1^ Also, the optimal timing of oral anticoagulation after ischemic stroke in atrial fibrillation is an unresolved clinical challenge.^23^ As we have shown that most sICHs occur within 12 h after EVT and less frequently after more than 24 h, it may be safe to start oral anticoagulation earlier than advised in the AHA/ASA guidelines.^1^ This could potentially reduce the risk of early recurrent ischemic stroke.^22^ However, results of randomized controlled trials are needed to give more clarity concerning this issue.^23–25^

### Limitations

Our study has limitations. First, this was a retrospective study subjecting it to the potential biases inherent to this type of analysis. Second, as time of neurological deterioration was not documented, we had to use the examination time of first CT-scan on which ICH was found as a proxy. Initiation of sICH will have occurred earlier. However, as it is standard protocol in the Netherlands to immediately perform a CT-scan when neurological deterioration occurs, we expect only a short delay. Third, in 27/204 (13%) patients, timing of sICH could not be retrieved. This may have influenced the results. However, we consider it likely that these values were not associated to outcome (i.e. technical issues), limiting a potential influence. In addition, a sensitivity analysis including imputation of missing values for timing of sICH showed comparable results as the primary analysis based on complete cases. Fourth, some patients were lost during the follow-up period, potentially introducing a bias. However, as the majority of sICHs occurred during the first day and only a small number of patients (23/3391) were lost to follow-up within 1 day after EVT, we expect no influence on the results. Finally, the exclusion of patients receiving EVT outside a MR CLEAN trial center may affect generalizability. However, as we have national guidelines and quality requirements to which all EVT-centers in the Netherlands adhere we expect results would not have differed if these patients had been included.

## Conclusion

SICHs primarily occur in the first hours after endovascular stroke treatment, and much less frequently beyond 24 h. Stroke guidelines that advise to perform frequent neurological assessments for at least 24 h after intravenous alteplase treatment should be applied to ischemic stroke patients treated with EVT.

## Supplemental Material

sj-docx-1-eso-10.1177_23969873221112279 – Supplemental material for Timing of symptomatic intracranial hemorrhage after endovascular stroke treatment Click here for additional data file. Supplemental material, sj-docx-1-eso-10.1177_23969873221112279 for Timing of symptomatic intracranial hemorrhage after endovascular stroke treatment by Wouter van der Steen, Nadinda AM van der Ende, Katinka R van Kranendonk, Vicky Chalos, Josje Brouwer, Robert J van Oostenbrugge, Wim H van Zwam, Pieter J van Doormaal, Adriaan CGM van Es, Charles BLM Majoie, Aad van der Lugt, Diederik WJ Dippel and Bob Roozenbeek in European Stroke Journal
