# Supplementary material for: Timing of symptomatic intracranial hemorrhage after endovascular stroke treatment
Source: Eur Stroke J. 2022 Aug 3;7(4):393–401. doi: 10.1177/23969873221112279 (PMC9720857; doi:10.1177/23969873221112279)
Supplement: sj-docx-1-eso-10.1177_23969873221112279 – Supplemental material for Timing of symptomatic intracranial hemorrhage after endovascular stroke treatment [file sj-docx-1-eso-10.1177_23969873221112279.docx]

SUPPLEMENTAL MATERIAL ON:

**TIMING OF SYMPTOMATIC INTRACRANIAL HEMORRHAGE AFTER ENDOVASCULAR STROKE TREATMENT**

*Wouter van der Steen, MD^1,2^; Nadinda A.M. van der Ende, MD^1,2^; Katinka R. van Kranendonk, MD^3^; Vicky Chalos, MD^1,2,4^; J. Brouwer, MD^5^; Robert J. van Oostenbrugge, MD, PhD^6^; Wim H. van Zwam, MD, PhD^7^; Pieter J. van Doormaal, MD^1^; Adriaan C.G.M. van Es, MD, Ph^8^; Charles B.L.M. Majoie, MD, PhD^3^; Aad van der Lugt, MD, PhD^2^; Diederik W.J. Dippel, MD, PhD^1^; Bob Roozenbeek, MD, PhD^1,2^; on behalf of the MR CLEAN trial and MR CLEAN Registry investigators*

**Affiliations:**

1. Department of Neurology, Erasmus MC University Medical Center, Rotterdam, The Netherlands
2. Department of Radiology & Nuclear Medicine, Erasmus MC University Medical Center, Rotterdam, The Netherlands
3. Department of Radiology & Nuclear Medicine, Amsterdam University Medical Center, University of Amsterdam, Amsterdam, The Netherlands
4. Department of Public Health, Erasmus MC University Medical Center, Rotterdam, The Netherlands
5. Department of Neurology, Amsterdam University Medical Centers, University of Amsterdam, Amsterdam, The Netherlands
6. Department of Neurology, Maastricht University Medical Center, Maastricht, The Netherlands
7. Department of Radiology & Nuclear Medicine, Maastricht University Medical Center, Maastricht, The Netherlands
8. Department of Radiology, Leiden University Medical Center, Leiden, The Netherlands

| **Table of Contents** |  |
| --- | --- |
| Supplemental Methods…………………………………………………………………... | 3 |
| Sources of Data and Measurement of Covariates……………………………………. | 3 |
| Supplemental Tables…………………………………………………………………….. | 4 |
| Supplemental Table 1………………………………………………………………… | 4 |
| Supplemental Table 2………………………………………………………………… | 5 |
| Supplemental Investigator List…………………………………………………………... | 6 |
| MR CLEAN Registry Investigators………………………………………………….. | 6 |
| MR CLEAN Trial Investigators……………………………………………………… | 7 |
| List of affiliations…………………………………………………………………….. | 8 |

Supplemental Methods

**Sources of Data and Measurement of Covariates**

In both the MR CLEAN trial and the MR CLEAN Registry a trial office used case report forms to match patient data from electronic patients files with a specific study identification number. Using this number, all data were entered into a good-clinical-practice approved, web-based clinical database (OpenClinica Community). The treating interventional radiologist provided data related to the endovascular procedure through a separate case report form. The study coordinators of the studies checked all data for completeness, formatting, and consistency. To check on reporting of safety, all discharge letters were screened for complications including symptomatic intracranial hemorrhage. For patients transferred from a referring stroke center to an intervention center, clinical and imaging data from the referring stroke center were collected and stored centrally. An imaging core laboratory assessed the images. The members of this core laboratory were blinded to all clinical findings, with the exception of clinical assessment of the occlusion location in case of baseline non-contrast computed tomography. For the MR CLEAN trial, observers were also blinded for treatment allocation (thrombectomy vs. control). Before assessment began, the observers were provided with guidelines including relevant definitions. To reach a TICI score of 2B or higher, it was mandatory to complete digital subtraction angiography runs including anteroposterior and lateral views after endovascular treatment. If a lateral view was missing, 2A was the highest possible score. For this analysis, the first author (WvdS) evaluated all available scans of patients with sICH, and used the DICOM data to determine the examination time of first scan on which ICH was found. To be able to merge the data, we transformed the extended TICI of the MR CLEAN Registry cohort to the modified TICI score by transforming grade 2C scores to grade 2B scores.

Supplemental Tables

| **Supplemental Table 1.** Hemorrhage types found in patients with sICH, stratified by timing of sICH after endovascular stroke treatment | | | |
| --- | --- | --- | --- |
|  | Timing of sICH <24h (n=137) | Timing of sICH ≥24h (n=41) | Timing of sICH unknown* (n=27) |
| Isolated hemorrhagic infarction, n (%) | 2 (1.5) | 2 (4.9) | 1 (7.7) |
| Isolated parenchymal hematoma, n (%) | 29 (21) | 14 (34) | 2 (15) |
| Hemorrhage outside infarcted brain tissue, n (%) | 16 (12) | 3 (7.3) | 4 (31) |
| Combined hemorrhage, n (%) | 90 (66) | 22 (54) | 6 (46) |
| *Hemorrhage types are presented as frequencies (n) and percentages (%).*  **In 14 patients the hemorrhage type is also unknown, because follow-up imaging could not be assessed by core lab committee.* | | | |

| **Supplemental Table 2.** Univariable and multivariable regression analysis of determinants of timing of sICH after endovascular stroke treatment including imputation of missing values for time to sICH | | |
| --- | --- | --- |
| **Variables** | **β (95% CI)** | **Adjusted β (95% CI)** |
| Age (per 10 years) | 1.05 (0.80-1.38) | 0.91 (0.68-1.21) |
| Male sex | 1.02 (0.53-1.95) | 0.85 (0.43-1.69) |
| Medical history of stroke | 0.74 (0.33-1.64) | 0.57 (0.23-1.42) |
| Medical history of atrial fibrillation | 1.84 (0.85-3.98) | 1.53 (0.58-4.00) |
| Medical history of vascular disease | 1.20 (0.54-2.67) | 1.08 (0.45-2.58) |
| Prior use of antiplatelets | 0.91 (0.47-1.77) | 1.20 (0.57-2.51) |
| Prior use of anticoagulants | 1.84 (0.82-4.10) | 1.52 (0.47-4.98) |
| Baseline NIHSS (per point increase) | 1.03 (0.98-1.08) | 1.04 (0.99-1.10) |
| Baseline systolic blood pressure (per 10 mmHg) | 1.02 (0.91-1.14) | 1.02 (0.90-1.16) |
| Baseline glucose level (per mmol/L) | 1.00 (0.96-1.04) | 0.97 (0.93-1.02) |
| Baseline platelet count (per 10*10ˆ9/L) | 1.00 (0.96-1.03) | 1.00 (0.97-1.04) |
| Baseline INR (per point increase) | 1.98 (0.85-4.63) | 2.28 (0.66-7.87) |
| Baseline ASPECTS on NCCT (per point increase) | 0.91 (0.85-4.63) | 0.88 (0.75-1.04) |
| Poor collateral score (<50%) | 0.82 (0.44-1.53) | 0.74 (0.38-1.44) |
| Intravenous alteplase treatment | 0.94 (0.47-1.90) | 1.49 (0.61-3.64) |
| Performed procedure (EVT vs. DSA or catheterization only) | 0.63 (0.24-1.61) | 0.52 (0.19-1.41) |
| Poor post-EVT mTICI score (<2B) | 0.85 (0.43-1.65) | 0.69 (0.34-1.38) |
| Onset to reperfusion time (per hour) | 1.00 (1.00-1.01) | 1.00 (1.00-1.01) |
| *Univariable and multivariable regression coefficients are presented as beta (β) coefficients with 95% confidence intervals (CI). sICH indicates symptomatic intracranial hemorrhage; NIHSS, National Institutes of Health Stroke Scale;* *INR, International Normalized Ratio; ASPECTS, Alberta Stroke Program Early CT score; NCCT, Non-Contrast CT;* *EVT, Endovascular Therapy; DSA, Digital Subtraction Angiography; and mTICI, modified Thrombolysis in Cerebral Infarction.* | | |

Supplemental Investigator List

## **Investigator list and affiliations of MR CLEAN Registry and MR CLEAN Trial Investigators**

## **MR CLEAN Registry Investigators**

Executive committee

Diederik W.J. Dippel^1^;Aad van der Lugt^2^;Charles B.L.M. Majoie^3^;Yvo B.W.E.M. Roos^4^;Robert J. van Oostenbrugge^5^;Wim H. van Zwam^6^;Jelis Boiten^14^;Jan Albert Vos^8^

Study coordinators

Ivo G.H. Jansen^3^;Maxim J.H.L. Mulder^1,2^;Robert- Jan B. Goldhoorn^5,6^;Kars C.J. Compagne^2^;Manon Kappelhof^3^;Josje Brouwer^4^;Sanne J. den Hartog^1,2,40^;Wouter H. Hinsenveld ^5,6^;

Local principal investigators

Diederik W.J. Dippel^1^;Bob Roozenbeek^1^;Aad van der Lugt^2^;Adriaan C.G.M. van Es^2^;Charles B.L.M. Majoie^3^;Yvo B.W.E.M. Roos^4^;Bart J. Emmer^3^;Jonathan M. Coutinho^4^;Wouter J. Schonewille^7^;Jan Albert Vos^8^; Marieke J.H. Wermer^9^;Marianne A.A. van Walderveen^10^;Julie Staals^5^;Robert J. van Oostenbrugge^5^;Wim H. van Zwam^6^;Jeannette Hofmeijer^11^;Jasper M. Martens^12^;Geert J. Lycklama à Nijeholt^13^;Jelis Boiten^14^;Sebastiaan F. de Bruijn^15^;Lukas C. van Dijk^16^;H. Bart van der Worp^17^;Rob H. Lo^18^;Ewoud J. van Dijk^19^;Hieronymus D. Boogaarts^20^;J. de Vries^22^;Paul L.M. de Kort^21^; Julia van Tuijl^21^ ; Jo P. Peluso^26^;Puck Fransen^22^;Jan S.P. van den Berg^22^;Boudewijn A.A.M. van Hasselt^23^;Leo A.M. Aerden^24^;René J. Dallinga^25^;Maarten Uyttenboogaart^28^;Omid Eschgi^29^;Reinoud P.H. Bokkers^29^;Tobien H.C.M.L. Schreuder^30^;Roel J.J. Heijboer^31^;Koos Keizer^32^;Lonneke S.F. Yo^33^;Heleen M. den Hertog^22^;Tomas Bulut^35^; Paul J.A.M. Brouwers^34^

Imaging assessment committee

Charles B.L.M. Majoie^3^(chair);Wim H. van Zwam^6^;Aad van der Lugt^2^;Geert J. Lycklama à Nijeholt^13^;Marianne A.A. van Walderveen^10^;Marieke E.S. Sprengers^3^;Sjoerd F.M. Jenniskens^27^;René van den Berg^3^;Albert J. Yoo^38^;Ludo F.M. Beenen^3^;Alida A. Postma^6^;Stefan D. Roosendaal^3^;Bas F.W. van der Kallen^13^;Ido R. van den Wijngaard^13^;Adriaan C.G.M. van Es^2^;Bart J. Emmer^,3^;Jasper M. Martens^12^; Lonneke S.F. Yo^33^;Jan Albert Vos^8^; Joost Bot^36^, Pieter-Jan van Doormaal^2^; Anton Meijer^27^;Elyas Ghariq^13^; Reinoud P.H. Bokkers^29^;Marc P. van Proosdij^37^;G. Menno Krietemeijer^33^;Jo P. Peluso^26^;Hieronymus D. Boogaarts^20^;Rob Lo^18^;Wouter Dinkelaar^2^Auke P.A. Appelman^29^;Bas Hammer^16^;Sjoert Pegge^27^;Anouk van der Hoorn^29^;Saman Vinke^20^.

Writing committee

Diederik W.J. Dippel^1^(chair);Aad van der Lugt^2^;Charles B.L.M. Majoie^3^;Yvo B.W.E.M. Roos^4^;Robert J. van Oostenbrugge^5^;Wim H. van Zwam^6^;Geert J. Lycklama à Nijeholt^13^;Jelis Boiten^14^;Jan Albert Vos^8^;Wouter J. Schonewille^7^;Jeannette Hofmeijer^11^;Jasper M. Martens^12^;H. Bart van der Worp^17^;Rob H. Lo^18^

Adverse event committee

Robert J. van Oostenbrugge^5^(chair);Jeannette Hofmeijer^11^;H. Zwenneke Flach^23^

Trial methodologist

Hester F. Lingsma^40^

Research nurses / local trial coordinators

Naziha el Ghannouti^1^;Martin Sterrenberg^1^;Wilma Pellikaan^7^;Rita Sprengers^4^;Marjan Elfrink^11^;Michelle Simons^11^;Marjolein Vossers^12^;Joke de Meris^14^;Tamara Vermeulen^14^;Annet Geerlings^19^;Gina van Vemde^22^;Tiny Simons^30^;Gert Messchendorp^28^;Nynke Nicolaij^28^;Hester Bongenaar^32^;Karin Bodde^24^;Sandra Kleijn^34^;Jasmijn Lodico^34^; Hanneke Droste^34^;Maureen Wollaert^5^;Sabrina Verheesen^5^;D. Jeurrissen^5^;Erna Bos^9^;Yvonne Drabbe^15^;Michelle Sandiman^15^;Nicoline Aaldering^11^;Berber Zweedijk^17^;Jocova Vervoort^21^;Eva Ponjee^22^;Sharon Romviel^19^;Karin Kanselaar^19^;Denn Barning^10^.

PhD / Medical students

Esmee Venema^40^; Vicky Chalos^1,40^; Ralph R. Geuskens^3^; Tim van Straaten^19^;Saliha Ergezen^1^; Roger R.M. Harmsma^1^; Daan Muijres^1^; Anouk de Jong^1^;Olvert A. Berkhemer^1,3,6^;Anna M.M. Boers^3,39^; J. Huguet^3^;P.F.C. Groot^3^;Marieke A. Mens^3^;Katinka R. van Kranendonk^3^;Kilian M. Treurniet^3^;Manon L. Tolhuisen^3,39^;Heitor Alves^3^;Annick J. Weterings^3^,Eleonora L.F. Kirkels^3^,Eva J.H.F. Voogd^11^;Lieve M. Schupp^3^;Sabine L. Collette^28,29^;Adrien E.D. Groot^4^;Natalie E. LeCouffe^4^;Praneeta R. Konduri^39^;Haryadi Prasetya^39^;Nerea Arrarte-Terreros^39^;Lucas A. Ramos^39^.

## **MR CLEAN Trial investigators**

Olvert A. Berkhemer, M.D.,^*,1,3^ Puck S.S. Fransen, M.D.,^*,1,2^ Debbie Beumer, M.D.,^*1,5^ Lucie A.

van den Berg, M.D.,^4^ Hester F. Lingsma, M.D., Ph.D.,^40^ Albert J. Yoo, M.D.,^41^ Wouter J.

Schonewille, M.D.,^7^ Jan Albert Vos, M.D., Ph.D.,^8^ Paul J. Nederkoorn, M.D., Ph.D.,^4^ Marieke

J.H. Wermer, M.D., Ph.D.,^9^ Marianne A.A. van Walderveen, M.D., Ph.D.,^10^ Julie Staals, M.D.,

Ph.D.,^5^ Jeannette Hofmeijer, M.D., Ph.D.,^11^ Jacques A. van Oostayen, M.D., Ph.D.,^12^ Geert J.

Lycklama à Nijeholt, M.D., Ph.D.,^13^ Jelis Boiten, M.D., Ph.D.,^14^ Patrick A. Brouwer, M.D.,^2^ Bart

J. Emmer, M.D., Ph.D.,^2^ Sebastiaan F. de Bruijn, M.D., Ph.D.,^15^ Lukas C. van Dijk, M.D.,^16^ L.

Jaap Kappelle, M.D., Ph.D.,^17^ Rob H. Lo, M.D.,^18^ Ewoud J. van Dijk, M.D., Ph.D.,^19^ Joost de

Vries, M.D., Ph.D.,^20^ Paul L.M. de Kort, M.D., Ph.D.,^21^ Willem Jan J. van Rooij, M.D., Ph.D.,^26^

Jan S.P. van den Berg, M.D., Ph.D.,^22^ Boudewijn A.A.M. van Hasselt, M.D.,^23^ Leo A.M. Aerden, M.D., Ph.D.,^24^ René J. Dallinga, M.D.,^25^ Marieke C. Visser, M.D., Ph.D.,^42^ Joseph C.J. Bot, M.D., Ph.D.,^36^ Patrick C. Vroomen, M.D., Ph.D.,^28^ Omid Eshghi, M.D.,^29^ Tobien H.C.M.L. Schreuder, M.D.,^30^ Roel J.J. Heijboer, M.D.,^31^ Koos Keizer, M.D., Ph.D.,^32^ Alexander V. Tielbeek, M.D., Ph.D.,^33^ Heleen M. den Hertog, M.D., Ph.D.,^34^ Renske M. van den Berg-Vos, M.D., Ph.D.,^43^ Giorgos B. Karas, M.D.,^44^ Ewout W. Steyerberg, M.D., Ph.D.,^40^ H. Zwenneke Flach, M.D.,^23^ Henk A. Marquering Ph.D.,^3,39^ Marieke E.S. Sprengers, M.D., Ph.D.,^3^ Sjoerd F.M. Jenniskens, M.D., Ph.D.,^27^ Ludo F.M. Beenen, M.D.,^3^ René van den Berg, M.D., Ph.D.,^3^ Peter J. Koudstaal, M.D., Ph.D.,^1^ Wim H. van Zwam, M.D., Ph.D.,^#,6^ Yvo B.W.E.M. Roos, M.D., Ph.D.,^#,4^ Aad van der Lugt, M.D., Ph.D.,^#,2^ Robert J. van Oostenbrugge, M.D., Ph.D.,^#,5^ Charles B.L.M. Majoie, M.D., Ph.D.,^#,3^ and Diederik W.J. Dippel, M.D., Ph.D.^#,1^

* Berkhemer, Fransen and Beumer contributed equally.

# van Zwam, Roos, van der Lugt, van Oostenbrugge, Majoie and Dippel contributed equally.

Data monitoring and safety board

Chair: Martin M. Brown, National Hospital for Neurology & Neurosurgery, London,

UK. Member: Thomas Liebig, Med. Fakultat, Univ Koln, Germany, Independent Statistician:

Theo Stijnen, Leiden University Medical Center, Leiden, the Netherlands

Advisory board

Tommy Andersson, neuro interventionist, Karolinska Univeristy Hospital, Stockholm,

Sweden, Heinrich Mattle, neurologist, University hospital, Bern, Switzerland, Nils Wahlgren,

neurologist, Karolinska Hospital, Stockholm, Sweden.

Research nurses / local trial coordinators

Esther van der Heijden, Naziha Ghannouti; Erasmus MC University Medical Center

Rotterdam, the Netherlands. Nadine Fleitour, Imke Hooijenga; Academic Medical Center

Amsterdam, the Netherlands. Corina Puppels, Wilma Pellikaan; Sint Antonius Hospital,

Nieuwegein, the Netherlands. Annet Geerling; Radboud University Nijmegen Medical

Center, the Netherlands. Annemieke Lindl-Velema; Maastricht University Medical Center,

the Netherlands. Gina van Vemde; Isala Klinieken, Zwolle, The Netherlands. Ans de Ridder,

Paut Greebe, University Medical Center Utrecht, the Netherlands. Jose de Bont-

Stikkelbroeck, Sint Elisabeth Hospital, Tilburg, the Netherlands. Joke de Meris, MC

Haaglanden, the Hague, the Netherlands. Kirsten Janssen, Leiden University Medical Center,

the Netherlands. Willy Struijk, HAGA Hospital, the Hague, the Netherlands.

PhD / Medical students

Silvan Licher, Nikki Boodt, Adriaan Ros, Esmee Venema, Ilse Slokkers, Raymie-Jayce Ganpat,

Maxim Mulder, Nawid Saiedie, Alis Heshmatollah, Stefanie Schipperen, Stefan Vinken,

Tiemen van Boxtel, Jeroen Koets; Erasmus MC University Medical Center Rotterdam, the

Netherlands. Merel Boers, Emilie Santos, Jordi Borst, Ivo Jansen, Manon Kappelhof, Marit Lucas, Ralph

Geuskens, Renan Sales Barros, Roeland Dobbe, Marloes Csizmadia; Academic Medical

Center Amsterdam, the Netherlands.

## **List of affiliations**

Department of Neurology^1^, Radiology^2^, Public Health^40^, Erasmus MC University Medical Center;

Department of Radiology and Nuclear Medicine^3^, Neurology^4^, Biomedical Engineering & Physics^39^, Amsterdam UMC, University of Amsterdam, Amsterdam;

Department of Neurology^5^, Radiology^6^, Maastricht University Medical Center and Cardiovascular Research Institute Maastricht (CARIM);

Department of Neurology^7^, Radiology^8^, Sint Antonius Hospital, Nieuwegein;

Department of Neurology^9^, Radiology^10^, Leiden University Medical Center;

Department of Neurology^11^, Radiology^12^, Rijnstate Hospital, Arnhem;

Department of Radiology^13^, Neurology^14^, Haaglanden MC, the Hague;

Department of Neurology^15^, Radiology^16^, HAGA Hospital, the Hague;

Department of Neurology^17^, Radiology^18^, University Medical Center Utrecht;

Department of Neurology^19^, Neurosurgery^20^, Radiology^27^, Radboud University Medical Center, Nijmegen;

Department of Neurology^21^, Radiology^26^, Elisabeth-TweeSteden ziekenhuis, Tilburg;

Department of Neurology^22^, Radiology^23^, Isala Klinieken, Zwolle;

Department of Neurology^24^, Radiology^25^, Reinier de Graaf Gasthuis, Delft;

Department of Neurology^28^, Radiology^29^, University Medical Center Groningen;

Department of Neurology^30^, Radiology^31^, Atrium Medical Center, Heerlen;

Department of Neurology^32^, Radiology^33^, Catharina Hospital, Eindhoven;

Department of Neurology^34^, Radiology^35^, Medisch Spectrum Twente, Enschede;

Department of Radiology^36^, Neurology^42^, Amsterdam UMC, Vrije Universiteit van Amsterdam, Amsterdam;
Department of Radiology^37^, Noordwest Ziekenhuisgroep, Alkmaar;

Department of Radiology^38^, Texas Stroke Institute, Texas, United States of America.

Department of Radiology^41^, Massachusetts General Hospital, Boston, United States of America;

Department of Neurology^43^, Radiology^44^, Sint Lucas Andreas Hospital, Amsterdam, the Netherlands;
